# Cryptic marine gastropods in Hawai’i exhibit variable response to multidecadal *in situ* environmental changes

**DOI:** 10.1371/journal.pone.0347347

**Published:** 2026-05-06

**Authors:** McClaran Shirley, Amber Dawn Stubler

**Affiliations:** 1 Biology Department, Occidental College, 1600 Campus Road, Los Angeles, California, United States of America; 2 Scripps Institution of Oceanography, UC San Diego, San Diego, California, United States of America; Griffith University, AUSTRALIA

## Abstract

Natural history collections are a valuable tool to assess the effects of broad-scale and long-term environmental change on a diverse assemblage of species. Measurements from species that have been repeatedly collected over time within the same region provide an opportunity to infer morphological differences that have arisen as changes have occurred at the actual *in situ* rate of change. Using a natural history shell collection with a strong regional focus, we assessed morphological changes in marine gastropods collected from Waianae, Oahu, Hawai’i, USA from 1974–2007 to investigate the consequences of *in situ* changes in environmental conditions. We measured shell length and weight of 1,502 specimens representing 37 species and 19 families. Most species were cryptic, micro-shelled species (n = 25; 67.5% of species) and had a mean length of less than 5 mm. Regression and linear mixed model analyses of log-transformed weight-to-length ratio by collection year, found that the majority of species (30 out of 37) did not exhibit any temporal trend, while 7 species exhibited significant declines over time. Though the observational format of museum collection studies constrains the ability to establish causal relationships between morphometric shifts and environmental changes, these findings offer insight into the potential resiliency and vulnerability of these cryptic, non-charismatic gastropods over an ecologically relevant timescale.

## Introduction

As ocean conditions continue to change [1], it is imperative to assess organismal response through both experimentation and observational data. To date, the majority of our empirical understanding of the specific impacts of ocean changes, such as acidification or increased temperatures, on marine species has been derived from controlled experimental exposures. Experimental manipulations aimed at investigating the effects of environmental changes (e.g., temperature, pH) are typically conducted over relatively short time periods (days to months) for captive organisms in laboratory or *in situ* settings [2–5]. Over the last two decades, controlled experiments have described compelling causal relationships between environmental changes and organismal response, but exhibit the following limitations: 1) experimental studies are unable to encompass the multi-decadal time period over which changes generally occur *in situ*, 2) many do not investigate multiple environmental stressors in tandem, such as warming and acidification, due to the high number of replicates and complex design required, and 3) by adhering to experimental design best practices, studies purposefully eliminate confounding and covarying factors that occur when these changes occur *in situ* [6–8]. While these conditions are necessary and important for obtaining quality data from controlled experimental investigations, it complicates and limits our ability to understand how future conditions will impact a wide array of organisms in the marine environment, given that we cannot replicate the predicted changes over relevant time scales in experimental studies.

One approach that can bridge the limitations between experimental studies and understanding the actualized impacts of environmental change is the use of natural history collections. Collections that include specimens obtained from the same locations over multi-decadal time spans [9,10] can offer unique hindsight into the effects of environmental changes occurring *in situ* over relevant time frames. This approach is especially effective when robust and long-term environmental data (e.g., temperature, pH, salinity) have been reliably collected in the same region as the specimens. Together, these environmental data and an appropriately representative natural history collection, can provide valuable insights into long-term change over time [11] and provide baseline data for future studies [10]. Museum collections have been successfully used in the terrestrial and marine world to assess the impacts of environmental changes on a wide array of organisms [10,12–14]. Many studies using museum collection specimens have focused on a single species, often an ecologically dominant, commercially important, keystone, or ‘indicator’ species with an abundant record, with far fewer studies focused on cryptic or lesser-known species. Given the importance of biodiversity to ecosystem resilience and the varying patterns of species response to environmental change, interrogating a multitude of species from regionally focused museum collections can provide valuable retrospective insights into patterns across a greater diversity of organisms.

Marine mollusks, especially shelled gastropods, are inherently vulnerable to environmental change, particularly changes in temperature and carbonate chemistry (ocean acidification) [15] and tend to be well-represented in natural history and museum collections. Shelled gastropods are reliant on the formation of shells primarily composed of calcium carbonate (calcite, aragonite, or a combination) and therefore experience morphologic impacts to their shells when carbonate saturation states are lowered (e.g., due to rising *p*CO_2_ and lower pH), and precipitation of shell material is impaired [16]. Experimental studies have shown that increased temperatures and acidification negatively impact the growth, calcification, and metabolic processes in shelled gastropods across all life history stages; however, taxonomic variability is prevalent [17,18]. Impacts to shell morphology, such as shell size, density, and strength/thickness have been demonstrated in experimentally acidified conditions, especially if organisms are exposed during larval and early developmental stages, or if warming occurs concurrently [19–21]. When these types of environmental changes occur *in situ*, the integrated impacts on morphology can be well-preserved in shell collections and can provide a proving ground for many of the experimental studies that cannot accurately recreate the *in situ* rate of change in environmental conditions for these organisms, nor the potential confounding impacts that may co-occur as a result.

Here we use a natural history collection of mollusks to investigate the morphologic patterns and trends from specimens collected over the last several decades from Waianae, Oahu, Hawai’i through an observational framework. We chose to focus on organisms from this specific location because, 1) the collection has a robust array of specimens from Waianae, HI, and 2) the environmental changes in atmospheric CO_2_, seawater CO_2_, pH, and calcium carbonate saturation state (both for aragonite and calcite) in this region have been well-characterized for decades (NOAA/PMEL). Measurements of atmospheric carbon dioxide in Hawai’i have been reliably collected at the Mauna Loa Observatory since the 1950s [22], and data from the Hawaii Ocean Time Series (1988–2007), ALOHA station (22°45’ N, 158°W) show that from 1988–2002, seawater pH decreased by ~0.04 units and aragonite saturation decreased by ~0.2 [23–25], though mean annual sea surface temperatures did not experience a significant pattern of increase (beyond the typical seasonal pattern) [25].

The Cosman Shell Collection at Occidental College (CSCOC), includes over 117,000 mollusk specimens repeatedly collected by Dieter Cosman, a dedicated hobbyist shell collector. Importantly, CSCOC shells were often gathered indiscriminately as live specimens during collection events (e.g., SCUBA dive where every specimen encountered was collected, Van Veer grab, etc.), and there are detailed metadata for every specimen collection lot; these factors make the CSCOC a scientifically valuable resource, especially for temporal change research. Using the CSCOC, we evaluated morphological changes across a total of 37 species of marine gastropods collected around Waianae, Oahu, Hawai’i, USA from 1974–2007. By comparing morphometric measurements across specimens collected during this 33-year period, this study investigates the consequences of *in situ* changes in environmental conditions on cryptic shelled species.

## Materials and methods

### Specimen eligibility and inclusion

This study used specimens from the Cosman Shell Collection at Occidental College, which were collected in or around Waianae, Oahu, Hawai’i, USA (21°26’ N, 158°11’W) between 1974 and 2007. No permits were required to access and measure the museum specimens, and all specimens were donated from the private collection of Dieter Cosman. Cosman primarily collected live specimens through a variety of methods including SCUBA, sediment grabs, and snorkeling. The inclusion criteria for species required that they: 1) belong to Class Gastropoda, 2) were collected live, 3) have >2 lots from Waianae (i.e., specimens collected at the same location and time), 4) have a minimum 20-year timespan between the oldest and newest specimen lots, and 5) have at least 3 specimens that were collected both prior to and after 1990, which serves as the functional midpoint in the period of investigation. Each included species had lots ranging from 3 to 17, with a sample size per lot ranging from n = 1 to n = 96 (S1 Table). Each specimen lot had attached metadata that, at minimum, detailed the collection location and date; in some cases, additional metadata including depth, habitat type and other collection notes were available. After meeting these criteria, a total of 1,502 specimens across 37 species (19 families) were morphometrically analyzed from collection dates occurring over 33 years (Table 1).

**Table 1:** Summary of species included, sample sizes, and mean morphometric measurements per species.

Species	Sample Size (n)	Mean Length (mm)	Mean Dry Mass (mg)	Mean Width (mm)	Mean Weight-to-Length Ratio (mg/mm)
*Acteocina sandwicensis*	120	3.12	2.64	1.28	0.77
*Alcyna ocellata*	136	2.27	1.81	1.43	0.78
*Alcyna subangulata*	43	1.89	1.01	1.29	0.52
*Bittinella hiloensis*	13	3.26	3.12	1.34	0.95
*Bouchetriphora pallida*	24	3.93	2.99	1.13	0.73
*Carinapex minutissima*	26	2.37	1.68	1.01	0.69
*Casmaria erinaceus*	13	34.92	8,312.20	19.91	178.92
*Cautor similis*	18	3.40	3.28	1.21	0.84
*Cysticus sandwicensis*	86	2.03	1.50	1.22	0.73
*Evalea eclecta*	24	3.65	1.59	0.92	0.43
*Granulina vitrea*	65	1.51	0.71	1.00	0.46
*Hastula lanceata*	64	28.20	551.17	5.35	18.96
*Haurakia marmorata*	65	1.51	0.49	0.88	0.32
*Herviera gliriella*	9	1.58	0.59	0.70	0.33
*Hydatina amplustre*	38	15.06	255.11	10.02	16.60
*Imbricaria flammea*	11	7.96	50.18	3.06	5.63
*Liloa mongii*	16	7.89	26.35	3.50	2.70
*Malea pomum*	12	62.40	33,675.79	46.19	517.27
*Mareleptopoma kenneyi*	24	1.38	0.48	0.84	0.34
*Mastonia cingulifera*	28	4.93	10.81	1.78	2.09
*Microcollonia rubricincta*	89	1.18	1.34	1.38	1.05
*Myurella affinis*	78	17.87	333.77	4.74	14.97
*Pandalosia ephamilla*	56	2.13	0.65	0.81	0.30
*Psilaxis oxytropis*	23	3.61	23.21	3.03	3.12
*Rissoina ambigua*	79	4.33	7.56	1.67	1.69
*Seminella virginea*	19	3.42	3.66	1.49	1.05
*Simulamerelina granulosa*	12	2.03	1.12	0.99	0.54
*Strigatella pudica*	67	13.73	444.56	7.02	30.98
*Styloptygma lacteolum*	14	4.86	4.55	1.32	0.83
*Subulophora peasi*	43	4.22	2.85	1.04	0.64
*Synaptocochlea concinna*	50	2.28	1.45	1.58	0.60
*Terebra guttata*	19	39.22	2,184.77	7.23	33.77
*Tridentarius dentatus*	59	28.30	1,492.50	10.05	46.64
*Turbonilla thaanumi*	13	2.42	0.70	0.74	0.28
*Turbonilla varicosa*	10	6.80	17.81	1.83	1.70
*Vexillum micra*	12	6.25	28.78	2.50	3.73
*Zafra smithi*	29	3.38	3.44	1.33	0.99

### Shell morphometrics

Standardized morphometric measurements of shell length and width, and dry weight were taken for each shell included in the study. Measurements were taken under a dissecting scope using a digital caliper (World Precision Instruments; 0.01 mm accuracy). Specimens were confirmed to be intact, and free from sediment or biological material before dry weights were obtained with an analytic balance (Sartorius A102S; 0.01 mg accuracy). Shell weight-to-length ratios (hereafter, WLR) were calculated using the dry weight (mg) and the shell length (longest axis, mm); WLR values were log-transformed for statistical analysis. Additionally, a representative specimen from each species was analyzed using the Horiba ExploRa + 203 dispersive Raman spectroscopy laser (532 nm) for shell mineral composition at the Natural History Museum of Los Angeles County, which confirmed that all species had aragonite shells.

### Statistical analyses

All statistical analyses were performed in R (ver. 4.5.1) [26], and an alpha = 0.05 was used (for code and data refer to S1-S3 Files). Since shell formation integrates the effects of all environmental conditions throughout the growth period, we used collection year as the predictor variable in our analyses rather than any single environmental variable. To determine whether the relationship between dry shell weight and length (WLR) changed over time for each species, the WLR was first log-transformed (log WLR, hereafter), and then we assessed temporal trends using either ordinary least squares (OLS) regressions or linear mixed-effects models (LMM). Given the potential non-independence of specimens within the same lot (pseudoreplication), we opted for a stratified approach where the analysis used for each species (OLS or LMM) was selected based on the degree of within-lot clustering, quantified by the intraclass correlation coefficient (ICC) [27]. The ICC represents the proportion of total variance in the log WLR attributable to lot-level grouping. Higher values indicate that specimens within the same lot are more similar to each other than to specimens from other lots (greater potential for non-independence), while species with lower ICC values typically had fewer specimens per lot and therefore had minimal within-lot clustering. We note that if environmental conditions were changing over time, then presumably lots collected within the same decade might be more similar to each other than to other lots, therefore higher ICC values may not necessarily indicate only non-independence in our data. We used an *a priori* ICC threshold of ≥0.15 [27,28] to account for the possibility that directional environmental change over the study period may have contributed to within-decade lot similarity independent of collection artefacts.

We first fit a preliminary linear mixed-effects model (LMM) with lot as a random intercept for all 37 species to obtain ICC values. For species that exceeded our *a priori* determined ICC threshold of ≥0.15 (n = 23 species), the within-lot clustering suggested that the use of an LMM approach was appropriate, and we fit the following model for each species: log WLR ~ year + (1 | lot), where lot was a random intercept. For species with ICC values <0.15 (n = 14 species), the within-lot clustering was considered negligible and OLS regressions (linear model: log WLR ~ year) were used since applying LMM to these species would reduce statistical power without any improvement to inference. A comparison of the OLS and LMM outputs for each species is provided in the S2 Table.

Additionally, this stratified approach was used to evaluate shell length changes over time. Using the same criteria, species were analyzed with either OLS or LMMs of log-transformed shell length (log L) over time (with lot as a random intercept in the LMMs) (S3 Table). These models were used to explore the relationship between body size (log L) and environmental change [29,30]; this analysis determined whether changes in log WLR were accompanied by significant declines in log L, which indicates weight changes were the driver of changes in WLR declines. For species that showed declines in both log WLR and log L, we calculated the ratio of the log WLR slope to the log L slope; a ratio greater than 1 indicates that log WLR is declining faster than log L (i.e., shell weight relative to length is changing more rapidly than shell length alone). We did not apply family-wise corrections (e.g., Bonferroni, Benjamini-Hochberg) to the species regressions (log WLR or log L), since each test represents an independent hypothesis about a biologically distinct species and the assumption of exchangeable null hypotheses underlying these corrections is not appropriate here [31,32]. The conservative nature of the stratified ICC-based approach provides meaningful protection against inflated false positive rates without imposing this assumption.

Following Tseng et al. [14] (and references within), we then transformed the slopes from each regression model to *‘% change in WLR per year’* using the equation (exp^(slope)^-1)*100 [14,29,33], where slope was the species-specific year slope from the stratified analysis. Using these values, we conducted a Standardized Major Axis regression (SMA; sometimes called Reduced Major Axis regression) to determine whether mean log WLR for each species was related to % change in WLR per year [34]. This analysis interrogates whether there is a relationship between WLR and amount of change per year experienced by each species (e.g., do species with a lower baseline WLR experience disproportionately greater rates of change in WLR over time?) [14,34]. This approach assumes equal relative (i.e., proportional) error variance and is typically preferred when the two variables are on different scales such as our data (log WLR and % change per year) [34]. The SMA regression was fit using the smatr package in R [35].

Since both mean log WLR and % change in WLR per year are derived from the same underlying weight and length measurements, this creates a risk that shared measurement error produces mathematical coupling. To evaluate this, we performed a permutation test to break the biological relationship between these values by shuffling species labels on % change in WLR per year while preserving the mathematical structure of both variables. A permutation test (n = 9,999) confirmed that the observed slope fell at the center of the null distribution, providing no evidence that mathematical coupling between the two variables influenced the result.

## Results

### Shell morphometrics

In total, 1,502 gastropod specimens from 37 species (19 families) were measured from the CSCOC; sample sizes within each species ranged from 7 to 136 samples with a mean sample size of 40.6 specimens per species (median = 26 specimens). Mean length of the 37 species ranged from 1.2 mm to 61.2 mm, though most species are considered cryptic, micro-shelled species (n = 25; 67.5% of species) with a mean length of less than 5 mm (see Table 1 for full morphometric summary).

### Linear regression models

**Fig 1:**
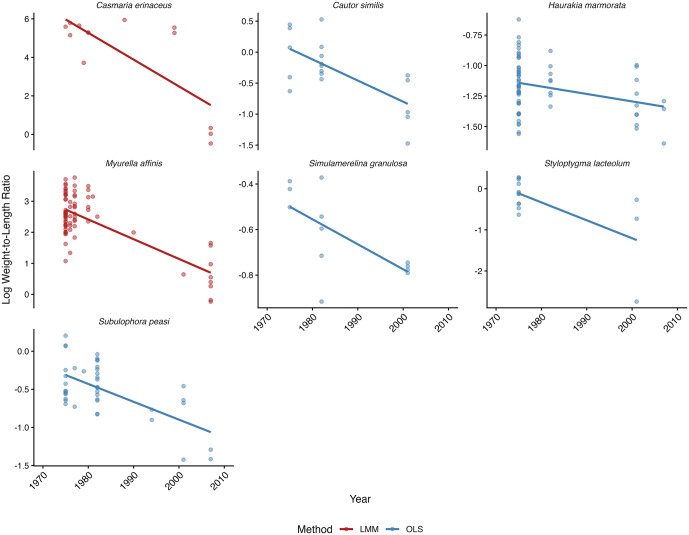
Significant Regression Models for 7 species. Species that showed significant relationships between year and log WLR are shown. All 7 species experienced a significant decline in log WLR (negative slope) over time. If a species was analyzed with OLS it is depicted with a solid dark blue line, whereas species analyzed with LMM are a solid dark red line.

**Fig 2:**
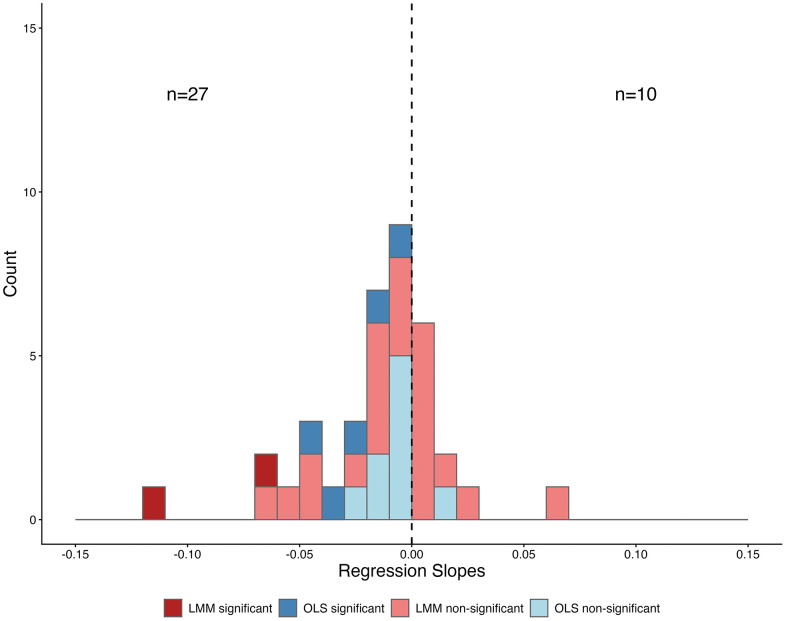
Histogram of slopes. Slopes from individual species regression analyses are represented in a histogram. Of 37 species, 27 of the slopes were negative (n = 7 significant), and 10 were positive (none were significant). The method of regression (LMM vs OLS) is indicated by color with OLS in blue and LMM in red, significant *P*-values from the regression are displayed by a darker shade of either blue or red.

For species with ICC ≥ 0.15, linear mixed-effects models (LMM) with lot as a random intercept (log WLR ~ year + 1 | lot) were used to ensure temporal trends were estimated from independent collection events (lots). For species with ICC < 0.15, ordinary least squares (OLS) regressions of log WLR by year were used (log WLR ~ year). Using this stratified approach, 23 species were fit to LMM and 14 were analyzed using OLS regressions; 7 species were found to have significant changes in log WLR over time (Fig 1, Table 2). For both methods, the residuals were inspected to ensure appropriate use of linear models. A histogram of slopes was created to visualize the collective response of the organisms (Fig 2). Within the 7 species that showed a significant relationship, all had negative slopes indicating a decrease in WLR over time (Table 2). The remaining 30 species had no significant pattern in log WLR over time (Table 2).

**Table 2:** Summary of regression model output values of log WLR and log length ~ year, grouped by family (in grey boxes). Bolded species indicate a significant *P*-value (<0.05) in either or both metrics.

Species (Grouped by Family)	Method	Log WLR slope	Log WLR *P*-value	Log L slope	Log L *P*-value	Response pattern
**Amplustridae**						
*Hydatina amplustre*	LMM	0.0099	0.633	0.0076	0.650	Neither significant
**Architectonicidae**						
*Psilaxis oxytropis*	LMM	−0.0300	0.427	−0.0147	0.473	Neither significant
**Atyidae**						
*Liloa mongii*	LMM	−0.0443	0.285	−0.0388	0.253	Neither significant
**Family Cassidae**						
***Casmaria erinaceus***	**LMM**	**−0.1114**	**0.048**	**−0.0581**	**0.021**	**Both declining**
**Family Collonidae**						
*Microcollonia rubricincta*	LMM	−0.0165	0.371	−0.0106	0.352	Neither significant
**Family Columbellidae**						
*Seminella virginea*	OLS	−0.0038	0.577	0.0027	0.449	Neither significant
*Zafra smithi*	LMM	−0.0102	0.491	−0.0027	0.723	Neither significant
**Family Costellaridae**						
***Vexillum micra***	**LMM**	**−0.0677**	**0.053**	**−0.0425**	**0.048**	**Log L declining only**
**Family Cystiscidae**						
*Cysticus sandwicensis*	LMM	0.0008	0.892	−0.0016	0.167	Neither significant
**Family Granulinidae**						
*Granulina vitrea*	LMM	0.0079	0.642	0.0014	0.713	Neither significant
**Family Mitridae**						
*Imbricaria flammea*	LMM	−0.0562	0.147	−0.0263	0.203	Neither significant
*Strigatella pudica*	OLS	−0.0100	0.379	−0.0015	0.688	Neither significant
**Family Pyramidellidae**						
*Evalea eclecta*	OLS	−0.0003	0.862	−0.0024	0.075	Neither significant
*Herviera gliriella*	LMM	0.0264	0.339	0.0062	0.595	Neither significant
***Styloptygma lacteolum***	**OLS**	**−0.0436**	**0.015**	**−0.0212**	**0.018**	**Both declining**
*Turbonilla thaanumi*	OLS	−0.0032	0.626	0.0025	0.517	Neither significant
*Turbonilla varicosa*	LMM	−0.0443	0.233	−0.0306	0.186	Neither significant
**Family Rissoidae**						
*Bittinella hiloensis*	LMM	−0.0047	0.692	−0.0012	0.373	Neither significant
***Haurakia marmorata***	**OLS**	**−0.0061**	**0.014**	**−0.0059**	**<0.001**	**Both declining**
*Mareleptopoma kenneyi*	LMM	0.0062	0.636	−0.0036	0.773	Neither significant
*Pandalosia ephamilla*	LMM	0.0023	0.739	−0.0010	0.236	Neither significant
*Rissoina ambigua*	LMM	0.0101	0.319	0.0047	0.387	Neither significant
***Simulamerelina granulosa***	**OLS**	**−0.0110**	**0.017**	**−0.0012**	**0.805**	**Log WLR declining**
**Family Scaphandridae**						
*Acteocina sandwicensis*	LMM	−0.0117	0.628	−0.0052	0.641	Neither significant
**Family Strombidae**						
*Tridentarius dentatus*	OLS	0.0154	0.278	0.0070	0.183	Neither significant
**Family Terebridae**						
*Hastula lanceata*	LMM	0.0081	0.371	0.0074	0.291	Neither significant
***Myurella affinis***	**LMM**	**−0.0646**	**0.011**	**−0.0395**	**0.005**	**Both declining**
*Terebra guttata*	LMM	0.0664	0.083	0.0387	0.084	Neither significant
**Family Tonnoidae**						
***Malea pomum***	**OLS**	**−0.0207**	**0.202**	**−0.0193**	**0.049**	**Log L declining only**
**Family Triphoridae**						
*Bouchetriphora pallida*	LMM	−0.0028	0.894	−0.0006	0.951	Neither significant
***Cautor similis***	**OLS**	**−0.0341**	**0.002**	**−0.0311**	**<0.001**	**Both declining**
*Mastonia cingulifera*	OLS	−0.0113	0.206	−0.0071	0.178	Neither significant
***Subulophora peasi***	**OLS**	**−0.0234**	**<0.001**	**−0.0130**	**<0.001**	**Both declining**
**Family Trochidae**						
***Alcyna ocellata***	**LMM**	**−0.0136**	**0.196**	**−0.0106**	**0.017**	**Log L declining only**
*Alcyna subangulata*	LMM	−0.0060	0.512	−0.0044	0.278	Neither significant
***Synaptocochlea concinna***	**OLS**	**−0.0101**	**0.090**	**−0.0072**	**0.014**	**Log L declining only**
**Family Turridae**						
*Carinapex minutissima*	OLS	−0.0035	0.454	−0.0041	0.164	Neither significant

Of the 7 species showing significant temporal declines in log WLR (Fig 1), 6 also showed a significant decline in log-transformed shell length (log L), indicating concurrent reductions in both shell length (L) and shell weight-to-length ratios (WLR) over the study period (Table 2). Across the 6 species showing declines in both log L and WLR, the rate of decline in log WLR consistently exceeded that of log L (slope ratio range: 1.03–2.06), indicating that shell weight decreased disproportionately relative to length. The loss of shell material was not simply a consequence of smaller shell size but reflected an independent reduction in shell mass per unit length. One species (*Simulamerelina granulosa*) showed a significant decline in log WLR without a simultaneous decline in log L, suggesting that this species maintained linear shell growth while losing shell weight relative to size. Interestingly, there were 4 species that had a significant pattern of decline in log L but not WLR over time (Table 2).

Finally, the SMA regression analyses [14,34] showed that interspecific variation in WLR was not explained by species-specific mean log WLR in the linear model relating species-specific % change in WLR per year to mean log WLR (*P* = 0.3, R^2^ = 0.03), indicating that there is no relationship between the WLR and the change in WLR per year for a species.

## Discussion

We assessed morphological changes in marine gastropods collected from Waianae, Oahu, Hawai’i, USA from 1974–2007 to investigate the consequences of *in situ* changes in environmental conditions, including seawater absorption of anthropogenic CO_2_. Our data indicate a range of sensitivity to environmental change among the 37 species examined (n = 30 experienced no change, n = 7 had decreased log shell WLR, and n = 10 had decreased log shell length over time; Table 2). For most of the species investigated, there is little known about their ecological importance, interactions, and broader roles in the community, a knowledge gap that limits our ability to identify mechanistic explanations for the observed patterns. Regardless, this work provides a glimpse into the potential resiliency and susceptibility of these cryptic, ecologically non-dominant species within the marine community over a relevant timeframe.

Despite the overarching expectation that marine calcifying species will experience major physiological impacts as global environmental change occurs, most species did not exhibit a significant decrease in either log shell WLR over time or log shell length. This is consistent with growing evidence that the response of marine organisms to environmental change is context-dependent and difficult to predict from single-stressor or controlled lab experiments alone. Experimental work has shown that isolated stressors within controlled experiments (e.g., either temperature or acidification) produce a bigger impact than when they co-occur in more realistic or multi-stressor conditions [36]. This highlights the difficulty of extrapolating controlled experiments to *in situ* environments where organisms respond to many simultaneous changes. Since the species in our study experienced environmental changes *in situ*, it is possible that stressors were buffered by other changes happening in the environment (e.g., increased food availability). Additionally, the 33-year period of relatively gradual *in situ* changes may have allowed organisms to acclimate or adapt, resulting in fewer detectable impacts on shell morphology among less sensitive species. Because the most recent specimen collection event occurred in 2007, and the rate of environmental change has since accelerated [1], future work that includes specimens collected within the last two decades would strengthen our general understanding of how accelerating *in situ* environmental change impacts marine gastropods over meaningful ecological timescales.

Though 30 species did not exhibit a significant pattern of change in shell log WLR over time, 7 species experienced a significant decrease. This is notable given a null hypothesis of no change-over-time for each species, and it highlights the variability in how gastropods respond to shifting oceanographic conditions (Table 2). The log L analysis showed that 6 of the 7 species with significant WLR declines experienced concurrent declines in shell length. This suggests these species are more sensitive to environmental changes, given that both growth rate and shell weight were negatively impacted over time. There were 4 species that showed declines in log L, but not WLR (Table 2); this morphometric pattern suggests that while these species displayed a decline in length over time, the weight-to-length ratio is being preserved and there is not a disproportionate loss in weight as length changes. The mechanisms underlying this response may fundamentally differ from those driving concurrent log L and log WLR changes and may suggest an overall growth suppression occurring in these species. The variability likely reflects species-specific differences in physiology, life history, and ecological roles, and may indicate that certain species are better positioned to employ compensatory mechanisms to sustain shell formation under changing conditions. For most species in our study, there is a profound lack of biological and ecological information, so we cannot directly evaluate which traits might lead to sensitivity or resilience.

The variability in species response may reflect differences in the physiological mechanisms governing shell formation, which are sensitive to environmental conditions in complex and species-specific ways. Calcium carbonate shell production in marine organisms is primarily constrained by temperature, availability of carbonate (CO_3_^2-^) and calcium (Ca^2+^) ions, and the metabolic capabilities of the organism. Increasing temperature can have both positive and negative impacts on physiological processes and metabolism, depending on a species’ thermal tolerance and thermal history [37,38]. For organisms existing below their upper thermal tolerance limit, warming may result in increased metabolic demand, feeding and increased shell growth, but for organisms living at or near their upper thermal limit, warming negatively affects growth and energetic budget, especially if combined with other stressors like acidification [37,38]. In gastropods, shell formation is a biologically controlled process [38–40] that occurs in a closed internal chamber, and is therefore directly related to an organisms’ energy budget. Some species can actively regulate the ion concentration in the fluid space where shell formation occurs to create a favorable internal environment for shell mineralization even when carbonate chemistry and ambient ion concentrations are unfavorable, albeit at a greater metabolic cost to the organism [17]. Though this may allow some species to continue to produce shells unabated during unfavorable conditions, the increased metabolic investment may require the organism to divert energy from other activities (e.g., reproduction, organic body growth [41]) or otherwise compensate (e.g., by consuming more food, expending less energy searching for food). This complex relationship between metabolism, biological control of *in vivo* ion concentrations, and thermal tolerance may provide a potential explanation why some species in our study remained morphologically stable in relation to changes in environmental conditions over the 33-year period examined.

Beyond the physiological capacity for shell formation, nutritional state has been shown to be a key component in the ability of calcifiers to modulate shell formation under varying environmental conditions [42]. Food availability and quality have been shown to buffer the impacts of acidification in some mollusks. For example, both laboratory and field experiments have shown that the bivalve *Mytilus edulis* is more resilient to acidification when food supply is abundant [43,44]. In a study leveraging natural history collections of the intertidal gastropod species, *Nucella lapillus*, Mayk et al. [13] found that the modern shells were thicker than those of historical specimens collected over a 130-year period and hypothesized that improvements in nutritional state may have allowed *N. lapillus* to invest more energy into shell production, despite evidence of unfavorable calcifying conditions. It is worth noting that *N. lapillus* showed increased shell thickness [13], whereas our sensitive species had decreased shell WLR; however, the underlying principle that nutritional state can impact energetic capacity for shell formation is relevant in either direction. Though limited, the current body of literature supports the expectation that nutritional state is an important factor affecting shell production and the impact of nutritional state on an organism’s energy budget are inherently integrated into shell morphometric variables. Future work incorporating condition index, shell compositional analysis (ratio of inorganic to organic components), and capacity for nutritional compensation might give insight into the underlying morphological resilience in some of the marine gastropod species examined.

Beyond nutritional quality and quantity, metabolic rates vary across species due to the differences in size and growth rates. Size-dependent responses to environmental change have been recorded for mollusks, where increased temperatures lead to increased metabolic demand and have an outsized impact on smaller individuals [45]. Recent studies have provided evidence for size-dependent responses to acidification among mollusks [45,46]. Waldbusser et al. [46] found that in larval bivalve clams, smaller individuals were more susceptible to the impacts of pH decreases; though there is a confounding ontogenetic component to this, since smaller individuals were also at different developmental stages and have different calcification rates. In contrast, larger chitons experienced a more pronounced metabolic depression than smaller individuals when exposed to increased *p*CO_2_ [45], suggesting size-dependent effects are not uni-directional across taxa. In our study, the lack of a significant relationship between the mean WLR and the change in WLR per year (SMA regression), indicates that initial shell size or WLR did not predispose species to morphological change over time and does not indicate size-dependent responses to the environmental changes for the species investigated.

Overall, our results suggest that resilience to environmental changes in the ocean is complex and likely a result of many simultaneous factors working synergistically (e.g., nutritional state, ability to regulate calcifying environment, growth rate as larvae, juvenile and adult) [47]. For the 7 species that exhibited a significant change in WLR, the functional consequences of a declining shell WLR are not well understood, particularly for these cryptic gastropods. In general, lower WLR values, indicating relatively lighter shells given organism size, may be more susceptible to physical breakage or predation due to lower shell strength [17,48,49]. The ecological consequences of this will depend on the primary function of the shell for each species (e.g., defense, habitat, desiccation resistance) and the degree to which a reduced WLR will impact the fitness and survival of that organism. Chatzinikolaou et al. [50] found that the shell density of *Columbella rustica*, a confamilial of two of our study species (*Seminella virginea* and *Zafra smithi*), was negatively impacted by the synergistic effects of low pH and increased temperature in a controlled laboratory experiment. Similarly, Barclay et al. [51] found that acidification negatively impacted shell strength for two intertidal gastropods (*Nucella ostrina* and *Tegula funebralis*), even when other shell growth metrics were unaffected. A follow-up study found that differences in the microstructural crystal arrangement in shell layers between the species led to greater vulnerability for *T. funebralis* [52]. For the species in our study, it is unclear whether any analogous microstructural differences exist, though our analysis using the Horiba ExploRa + 203 dispersive Raman spectroscopy laser (532 nm) confirmed that all had aragonite-based shells. Assessing the influence of shell microstructure (fibrous vs. heterogeneous fibrous calcium carbonate layers) on species-specific resilience, as Barclay et al. [52] demonstrated experimentally, was beyond the scope of this study given the absence of controlled environmental manipulation

A limitation of this study, and any museum-based dataset, is that sampling effort was not balanced and standardized over time. For nearly all species, lot-level effort (number of lots, species per lot) was higher in the 1970s and 1980s (S4 Table). This represents a drift in sampling intensity toward the earlier portion of the timeframe investigated (1974–2007); however, it’s worth noting that unless there was a systematic collection of disproportionately heavier shells in the earlier decades over the later decades (which is not supported by the metadata), there is no reason to expect a consistent pattern of decline among the 7 species in the absence of a biological signal. The patterns observed in the subset of gastropod species presented here from the CSCOC reflect the analytical advantages of geographically constrained, carefully curated natural history datasets, which avoid many of the spatial, temporal, and taxonomic biases that limit ecological inference in large-scale biodiversity repositories [53]. While these properties enhance within-system resolution, they necessarily limit the generalizability of our findings beyond the Hawaiian gastropod species studied here, and broader inference should be drawn cautiously and in conjunction with independent regional and global assessments.

## Conclusions

Natural history collections can be a valuable tool to assess impacts on biodiverse assemblages of rare or cryptic species that are difficult to include in experimental studies. The lack of relevant literature for the cryptic gastropods in our study only highlights the value of museum specimens as research subjects, especially where organisms are rare or threatened in their current ecosystems. Accurately predicting and fully understanding the effects of broad-scale environmental change associated with increases in atmospheric CO_2_ is a difficult task given that the rate of change in seawater pH/carbonate chemistry is orders of magnitude different from experimental assessments of species response. Ongoing and expected changes in seawater carbonate chemistry will take decades to occur *in situ*, and experimental assessments are unable to capture the actual rate of change experienced by organisms. Additionally, our current understanding of how environmental change will impact marine organisms is largely limited to single-species experiments, and while these provide important physiological information for the species involved, these experiments do not integrate other ecosystem changes that may occur simultaneously (e.g., changes in food availability, changes in predator abundance or behavior, etc.) nor do they account for potential impacts on the diverse assemblage of calcifiers found in marine environments.

Predicting how environmental change will impact marine organisms is more complex than simply understanding the impacts in isolation. Natural history collections, especially those with a strong regional focus, often have specimens collected at similar locations over time and can provide an integrated synopsis of the concurrent changes occurring within the ecosystem, better capturing the potential resiliency or susceptibility of organisms and communities. These data can be used to assess how organisms respond to global environmental changes at the actual rate of change within an ecosystem. While the observational format of museum collection studies limits the ability to establish direct causal relationships, this comparison of results between both experimental and observational studies at the very least highlights the biological significance of the morphological changes over time as found in specimens of the Cosman Shell Collection at Occidental College. The trends observed here reflect the responses of a specific Hawaiian reef gastropod assemblage over a 33-year period, and future work incorporating specimens from additional regions and more recent collection years will be essential for determining how broadly these patterns apply across reef communities and beyond.

## Supporting information

S1 Table Lot size summary per species. (DOCX)

S2 Table Direct comparison of OLS and LMM results for log WLR ~ year across all 37 species. Bold rows = significant under final assigned method.
(DOCX)

S3 Table Direct comparison of OLS and LMM results for log L ~ year across all 37 species. Bold rows = significant under final assigned method.
(DOCX)

S4 Table Number of lots and specimens per species per decade. Zeros indicate no collection activity in that decade.
(DOCX)

S1 File R Markdown file for analysis. This file is an annotated .RMD file containing the analyses presented in this manuscript.
(RMD)

S2 File Full Dataset. This .csv file is the dataset that was analyzed in this study.
(CSV)

S3 File Metadata for Dataset. This .csv file is the associated metadata for the collected data used in this study.
(XLSX)
