# Supplementary material for: Cryptic marine gastropods in Hawai’i exhibit variable response to multidecadal in situ environmental changes
Source: PLoS One. 2026 May 6;21(5):e0347347. doi: 10.1371/journal.pone.0347347 (PMC13148702; doi:10.1371/journal.pone.0347347)
Supplement: S1 Table — (DOCX) [file pone.0347347.s001.docx]

S1 Table. Lot size summary per species.

| **Species** | **N lots** | **Mean specimens/lot** | **Median specimens/lot** | **Max specimens/lot** |
| --- | --- | --- | --- | --- |
| *Acteocina sandwicensis* | 7 | 17.1 | 2.0 | 96 |
| *Alcyna ocellata* | 7 | 19.4 | 5.0 | 74 |
| *Alcyna subangulata* | 4 | 10.8 | 11.0 | 20 |
| *Bittinella hiloensis* | 3 | 4.3 | 3.0 | 8 |
| *Bouchetriphora pallida* | 4 | 6.0 | 2.0 | 18 |
| *Carinapex minutissima* | 5 | 5.2 | 4.0 | 10 |
| *Casmaria erinaceus* | 9 | 1.4 | 1.0 | 3 |
| *Cautor similis* | 5 | 3.6 | 3.0 | 8 |
| *Cysticus sandwicensis* | 6 | 14.3 | 8.0 | 50 |
| *Evalea eclecta* | 4 | 6.0 | 4.0 | 14 |
| *Granulina vitrea* | 6 | 10.8 | 9.0 | 25 |
| *Hastula lanceata* | 10 | 6.4 | 1.0 | 33 |
| *Haurakia marmorata* | 4 | 16.2 | 8.5 | 45 |
| *Herviera gliriella* | 4 | 2.2 | 2.0 | 4 |
| *Hydatina amplustre* | 4 | 9.5 | 3.0 | 31 |
| *Imbricaria flammea* | 4 | 2.8 | 2.0 | 6 |
| *Liloa mongii* | 4 | 4.0 | 2.0 | 11 |
| *Malea pomum* | 6 | 1.2 | 1.0 | 2 |
| *Mareleptopoma kenneyi* | 5 | 4.8 | 1.0 | 14 |
| *Mastonia cingulifera* | 5 | 5.6 | 1.0 | 22 |
| *Microcollonia rubricincta* | 5 | 17.8 | 3.0 | 74 |
| *Myurella affinis* | 11 | 7.1 | 3.0 | 35 |
| *Pandalosia ephamilla* | 6 | 9.3 | 8.5 | 18 |
| *Psilaxis oxytropis* | 8 | 2.9 | 1.0 | 14 |
| *Rissoina ambigua* | 8 | 9.9 | 6.0 | 28 |
| *Seminella virginea* | 5 | 3.8 | 2.0 | 13 |
| *Simulamerelina granulosa* | 4 | 3.0 | 3.5 | 4 |
| *Strigatella pudica* | 13 | 5.2 | 1.0 | 24 |
| *Styloptygma lacteolum* | 3 | 4.7 | 3.0 | 8 |
| *Subulophora peasi* | 9 | 4.8 | 2.0 | 18 |
| *Synaptocochlea concinna* | 4 | 12.5 | 8.5 | 32 |
| *Terebra guttata* | 9 | 2.1 | 1.0 | 8 |
| *Tridentarius dentatus* | 17 | 3.5 | 1.0 | 27 |
| *Turbonilla thaanumi* | 4 | 3.2 | 3.5 | 5 |
| *Turbonilla varicosa* | 6 | 1.7 | 1.0 | 4 |
| *Vexillum micra* | 7 | 1.7 | 2.0 | 3 |
| *Zafra smithi* | 4 | 7.2 | 4.5 | 19 |
