# Supplementary material for: Cryptic marine gastropods in Hawai’i exhibit variable response to multidecadal in situ environmental changes
Source: PLoS One. 2026 May 6;21(5):e0347347. doi: 10.1371/journal.pone.0347347 (PMC13148702; doi:10.1371/journal.pone.0347347)
Supplement: S2 Table — Bold rows = significant under final assigned method. (DOCX) [file pone.0347347.s002.docx]

S2 Table. Direct comparison of OLS and LMM results for log WLR ~ year across all 37 species. Bold rows = significant under final assigned method.

| **Species** | **ICC** | **Final method** | **OLS slope** | **OLS**  ***P*-value** | **OLS sig.** | **LMM slope** | **LMM**  ***P*-value** | **LMM sig.** |
| --- | --- | --- | --- | --- | --- | --- | --- | --- |
| *Rissoina ambigua* | 0.6834 | LMM | 0.0099 | 0.0007 | true | 0.0101 | 0.3191 | false |
| *Acteocina sandwicensis* | 0.7114 | LMM | 0.0228 | 0.0011 | true | -0.0117 | 0.6283 | false |
| ***Cautor similis*** | **0.0248** | **OLS** | **-0.0341** | **0.0018** | **true** | **-0.0341** | **0.0505** | **false** |
| *Imbricaria flammea* | 0.6056 | LMM | -0.0563 | 0.0036 | true | -0.0562 | 0.1465 | false |
| *Liloa mongii* | 0.4741 | LMM | -0.0552 | 0.0049 | true | -0.0443 | 0.2854 | false |
| *Vexillum micra* | 0.5956 | LMM | -0.0708 | 0.0051 | true | -0.0677 | 0.0531 | false |
| *Terebra guttata* | 0.6358 | LMM | 0.0786 | 0.0056 | true | 0.0664 | 0.0830 | false |
| ***Styloptygma lacteolum*** | **0.1229** | **OLS** | **-0.0436** | **0.0146** | **true** | **-0.0453** | **0.1992** | **false** |
| *Turbonilla varicosa* | 0.8049 | LMM | -0.0459 | 0.0164 | true | -0.0443 | 0.2326 | false |
| *Alcyna ocellata* | 0.6767 | LMM | -0.0087 | 0.0169 | true | -0.0136 | 0.1959 | false |
| ***Simulamerelina granulosa*** | **0.0792** | **OLS** | **-0.0110** | **0.0173** | **true** | **-0.0111** | **0.1526** | **false** |
| *Bittinella hiloensis* | 0.7937 | LMM | -0.0076 | 0.0495 | true | -0.0047 | 0.6916 | false |
| *Microcollonia rubricincta* | 0.3633 | LMM | -0.0095 | 0.0788 | false | -0.0165 | 0.3712 | false |
| *Synaptocochlea concinna* |  | OLS | -0.0101 | 0.0897 | false | -0.0101 | 0.0897 | false |
| *Herviera gliriella* | 0.6169 | LMM | 0.0242 | 0.1530 | false | 0.0264 | 0.3387 | false |
| *Cysticus sandwicensis* | 0.2439 | LMM | -0.0032 | 0.1598 | false | 0.0008 | 0.8924 | false |
| *Alcyna subangulata* | 0.2254 | LMM | -0.0049 | 0.1647 | false | -0.0060 | 0.5117 | false |
| *Mareleptopoma kenneyi* | 0.8217 | LMM | -0.0043 | 0.1678 | false | 0.0062 | 0.6360 | false |
| *Malea pomum* |  | OLS | -0.0207 | 0.2022 | false | -0.0207 | 0.2022 | false |
| *Mastonia cingulifera* |  | OLS | -0.0113 | 0.2064 | false | -0.0113 | 0.2064 | false |
| *Tridentarius dentatus* | 0.0590 | OLS | 0.0154 | 0.2781 | false | 0.0123 | 0.4773 | false |
| *Psilaxis oxytropis* | 0.8698 | LMM | 0.0225 | 0.3120 | false | -0.0300 | 0.4268 | false |
| *Strigatella pudica* |  | OLS | -0.0100 | 0.3793 | false | -0.0100 | 0.3793 | false |
| *Carinapex minutissima* |  | OLS | -0.0035 | 0.4539 | false | -0.0035 | 0.4539 | false |
| *Seminella virginea* |  | OLS | -0.0038 | 0.5769 | false | -0.0038 | 0.5769 | false |
| *Bouchetriphora pallida* | 0.6175 | LMM | -0.0036 | 0.5976 | false | -0.0028 | 0.8940 | false |
| *Turbonilla thaanumi* | 0.0684 | OLS | -0.0032 | 0.6260 | false | -0.0028 | 0.7218 | false |
| *Hydatina amplustre* | 0.5768 | LMM | 0.0024 | 0.6837 | false | 0.0099 | 0.6330 | false |
| *Zafra smithi* | 0.5587 | LMM | -0.0016 | 0.6874 | false | -0.0102 | 0.4913 | false |
| *Pandalosia ephamilla* | 0.6686 | LMM | 0.0005 | 0.8311 | false | 0.0023 | 0.7389 | false |
| *Evalea eclecta* | 0.0583 | OLS | -0.0003 | 0.8619 | false | -0.0007 | 0.7995 | false |
| *Granulina vitrea* | 0.7751 | LMM | 0.0001 | 0.9859 | false | 0.0079 | 0.6416 | false |
| *Hastula lanceata* | 0.6298 | LMM | 0.0000 | 0.9992 | false | 0.0081 | 0.3713 | false |
| ***Myurella affinis*** | **0.2964** | **LMM** | **-0.0635** | **0.0000** | **true** | **-0.0646** | **0.0105** | **true** |
| ***Subulophora peasi*** | **0.1139** | **OLS** | **-0.0234** | **0.0000** | **true** | **-0.0236** | **0.0062** | **true** |
| ***Casmaria erinaceus*** | **0.9608** | **LMM** | **-0.1391** | **0.0026** | **true** | **-0.1114** | **0.0483** | **true** |
| ***Haurakia marmorata*** |  | **OLS** | **-0.0061** | **0.0144** | **true** | **-0.0061** | **0.0144** | **true** |
