# Supplementary material for: Cryptic marine gastropods in Hawai’i exhibit variable response to multidecadal in situ environmental changes
Source: PLoS One. 2026 May 6;21(5):e0347347. doi: 10.1371/journal.pone.0347347 (PMC13148702; doi:10.1371/journal.pone.0347347)
Supplement: S3 Table — Bold rows = significant under final assigned method. (DOCX) [file pone.0347347.s003.docx]

S3 Table. Direct comparison of OLS and LMM results for log L ~ year across all 37 species. Bold rows = significant under final assigned method.

| **Species** | **ICC** | **Final method** | **OLS slope** | **OLS**  ***P*-value** | **OLS sig.** | **LMM slope** | **LMM**  ***P*-value** | **LMM sig.** |
| --- | --- | --- | --- | --- | --- | --- | --- | --- |
| *Acteocina sandwicensis* | 0.7114 | LMM | 0.0121 | 0.0002 | true | -0.0052 | 0.6410 | false |
| ***Haurakia marmorata*** |  | **OLS** | **-0.0059** | **0.0004** | **true** | **-0.0058** | **0.1831** | **false** |
| *Liloa mongii* | 0.4741 | LMM | -0.0467 | 0.0011 | true | -0.0388 | 0.2527 | false |
| *Hydatina amplustre* | 0.5768 | LMM | 0.0082 | 0.0025 | true | 0.0076 | 0.6502 | false |
| *Alcyna subangulata* | 0.2254 | LMM | -0.0049 | 0.0028 | true | -0.0044 | 0.2776 | false |
| *Turbonilla varicosa* | 0.8049 | LMM | -0.0306 | 0.0057 | true | -0.0306 | 0.1857 | false |
| *Terebra guttata* | 0.6358 | LMM | 0.0455 | 0.0067 | true | 0.0387 | 0.0835 | false |
| *Mareleptopoma kenneyi* | 0.8217 | LMM | -0.0037 | 0.0072 | true | -0.0036 | 0.7733 | false |
| *Imbricaria flammea* | 0.6056 | LMM | -0.0256 | 0.0138 | true | -0.0263 | 0.2029 | false |
| ***Styloptygma lacteolum*** | **0.1229** | **OLS** | **-0.0212** | **0.0181** | **true** | **-0.0260** | **0.4075** | **false** |
| *Rissoina ambigua* | 0.6834 | LMM | 0.0032 | 0.0464 | true | 0.0047 | 0.3871 | false |
| ***Malea pomum*** |  | **OLS** | **-0.0193** | **0.0486** | **true** | **-0.0172** | **0.1163** | **false** |
| *Microcollonia rubricincta* | 0.3633 | LMM | -0.0051 | 0.0550 | false | -0.0106 | 0.3522 | false |
| *Evalea eclecta* | 0.0583 | OLS | -0.0024 | 0.0748 | false | -0.0024 | 0.0748 | false |
| *Pandalosia ephamilla* | 0.6686 | LMM | -0.0010 | 0.1051 | false | -0.0010 | 0.2362 | false |
| *Carinapex minutissima* |  | OLS | -0.0041 | 0.1644 | false | -0.0041 | 0.1644 | false |
| *Cysticus sandwicensis* | 0.2439 | LMM | -0.0016 | 0.1668 | false | -0.0016 | 0.1668 | false |
| *Mastonia cingulifera* |  | OLS | -0.0071 | 0.1775 | false | -0.0071 | 0.1775 | false |
| *Tridentarius dentatus* | 0.0590 | OLS | 0.0070 | 0.1832 | false | 0.0061 | 0.3539 | false |
| *Psilaxis oxytropis* | 0.8698 | LMM | 0.0145 | 0.2561 | false | -0.0147 | 0.4731 | false |
| *Bittinella hiloensis* | 0.7937 | LMM | -0.0012 | 0.3729 | false | -0.0012 | 0.3729 | false |
| *Seminella virginea* |  | OLS | 0.0027 | 0.4488 | false | 0.0031 | 0.4292 | false |
| *Turbonilla thaanumi* | 0.0684 | OLS | 0.0025 | 0.5168 | false | 0.0024 | 0.5921 | false |
| *Granulina vitrea* | 0.7751 | LMM | -0.0006 | 0.5538 | false | 0.0014 | 0.7127 | false |
| *Herviera gliriella* | 0.6169 | LMM | 0.0045 | 0.5563 | false | 0.0062 | 0.5946 | false |
| *Strigatella pudica* |  | OLS | -0.0015 | 0.6877 | false | -0.0015 | 0.6877 | false |
| *Simulamerelina granulosa* | 0.0792 | OLS | -0.0012 | 0.8047 | false | -0.0012 | 0.8304 | false |
| *Zafra smithi* | 0.5587 | LMM | 0.0006 | 0.8366 | false | -0.0027 | 0.7227 | false |
| *Hastula lanceata* | 0.6298 | LMM | 0.0006 | 0.8604 | false | 0.0074 | 0.2907 | false |
| *Bouchetriphora pallida* | 0.6175 | LMM | -0.0003 | 0.9475 | false | -0.0006 | 0.9509 | false |
| ***Myurella affinis*** | **0.2964** | **LMM** | **-0.0381** | **0.0000** | **true** | **-0.0395** | **0.0046** | **true** |
| ***Alcyna ocellata*** | **0.6767** | **LMM** | **-0.0093** | **0.0000** | **true** | **-0.0106** | **0.0168** | **true** |
| ***Cautor similis*** | **0.0248** | **OLS** | **-0.0311** | **0.0000** | **true** | **-0.0309** | **0.0105** | **true** |
| ***Subulophora peasi*** | **0.1139** | **OLS** | **-0.0130** | **0.0005** | **true** | **-0.0133** | **0.0097** | **true** |
| ***Casmaria erinaceus*** | **0.9608** | **LMM** | **-0.0694** | **0.0009** | **true** | **-0.0581** | **0.0205** | **true** |
| ***Vexillum micra*** | **0.5956** | **LMM** | **-0.0443** | **0.0046** | **true** | **-0.0425** | **0.0483** | **true** |
| ***Synaptocochlea concinna*** |  | **OLS** | **-0.0072** | **0.0137** | **true** | **-0.0072** | **0.0137** | **true** |
