# Supplementary material for: Cryptic marine gastropods in Hawai’i exhibit variable response to multidecadal in situ environmental changes
Source: PLoS One. 2026 May 6;21(5):e0347347. doi: 10.1371/journal.pone.0347347 (PMC13148702; doi:10.1371/journal.pone.0347347)
Supplement: S4 Table — Zeros indicate no collection activity in that decade. (DOCX) [file pone.0347347.s004.docx]

S4 Table. Number of lots and specimens per species per decade. Zeros indicate no collection activity in that decade.

|  | **Lots per decade** | | | | **Specimens per decade** | | | | | **Totals** | | |  |
| --- | --- | --- | --- | --- | --- | --- | --- | --- | --- | --- | --- | --- | --- |
| **Species** | **Lots 1970s** | **Lots 1980s** | **Lots 1990s** | **Lots 2000s** | | **Spec. 1970s** | **Spec. 1980s** | **Spec. 1990s** | **Spec. 2000s** | | **Total lots** | **Total spec.** | |
| *Acteocina sandwicensis* | 1 | 2 | 1 | 3 | | 96 | 18 | 2 | 4 | | 7 | 120 | |
| *Alcyna ocellata* | 3 | 1 | 1 | 2 | | 50 | 74 | 4 | 8 | | 7 | 136 | |
| *Alcyna subangulata* | 1 | 1 | 0 | 2 | | 20 | 7 | 0 | 16 | | 4 | 43 | |
| *Bittinella hiloensis* | 0 | 2 | 0 | 1 | | 0 | 10 | 0 | 3 | | 3 | 13 | |
| *Bouchetriphora pallida* | 0 | 1 | 1 | 2 | | 0 | 18 | 2 | 4 | | 4 | 24 | |
| *Carinapex minutissima* | 1 | 2 | 0 | 2 | | 4 | 11 | 0 | 11 | | 5 | 26 | |
| *Casmaria erinaceus* | 5 | 2 | 1 | 1 | | 5 | 3 | 2 | 3 | | 9 | 13 | |
| *Cautor similis* | 2 | 1 | 0 | 2 | | 5 | 8 | 0 | 5 | | 5 | 18 | |
| *Cysticus sandwicensis* | 2 | 1 | 1 | 2 | | 54 | 18 | 1 | 13 | | 6 | 86 | |
| *Evalea eclecta* | 1 | 1 | 0 | 2 | | 14 | 4 | 0 | 6 | | 4 | 24 | |
| *Granulina vitrea* | 2 | 2 | 1 | 1 | | 12 | 33 | 5 | 15 | | 6 | 65 | |
| *Hastula lanceata* | 4 | 2 | 2 | 2 | | 56 | 2 | 4 | 2 | | 10 | 64 | |
| *Haurakia marmorata* | 1 | 1 | 0 | 2 | | 45 | 8 | 0 | 12 | | 4 | 65 | |
| *Herviera gliriella* | 1 | 1 | 0 | 2 | | 2 | 1 | 0 | 6 | | 4 | 9 | |
| *Hydatina amplustre* | 1 | 0 | 2 | 1 | | 5 | 0 | 32 | 1 | | 4 | 38 | |
| *Imbricaria flammea* | 0 | 2 | 1 | 1 | | 0 | 7 | 3 | 1 | | 4 | 11 | |
| *Liloa mongii* | 0 | 2 | 1 | 1 | | 0 | 12 | 2 | 2 | | 4 | 16 | |
| *Malea pomum* | 1 | 2 | 3 | 0 | | 2 | 2 | 3 | 0 | | 6 | 7 | |
| *Mareleptopoma kenneyi* | 1 | 1 | 1 | 2 | | 7 | 1 | 1 | 15 | | 5 | 24 | |
| *Mastonia cingulifera* | 2 | 2 | 0 | 1 | | 2 | 23 | 0 | 3 | | 5 | 28 | |
| *Microcollonia rubricincta* | 1 | 1 | 1 | 2 | | 74 | 1 | 3 | 11 | | 5 | 89 | |
| *Myurella affinis* | 5 | 3 | 1 | 2 | | 60 | 8 | 1 | 9 | | 11 | 78 | |
| *Pandalosia ephamilla* | 1 | 1 | 1 | 3 | | 1 | 18 | 12 | 25 | | 6 | 56 | |
| *Psilaxis oxytropis* | 2 | 4 | 0 | 2 | | 16 | 4 | 0 | 3 | | 8 | 23 | |
| *Rissoina ambigua* | 5 | 1 | 0 | 2 | | 29 | 20 | 0 | 30 | | 8 | 79 | |
| *Seminella virginea* | 1 | 1 | 1 | 2 | | 2 | 13 | 2 | 2 | | 5 | 19 | |
| *Simulamerelina granulosa* | 1 | 2 | 0 | 1 | | 3 | 5 | 0 | 4 | | 4 | 12 | |
| *Strigatella pudica* | 5 | 5 | 2 | 1 | | 49 | 15 | 2 | 1 | | 13 | 67 | |
| *Styloptygma lacteolum* | 2 | 0 | 0 | 1 | | 11 | 0 | 0 | 3 | | 3 | 14 | |
| *Subulophora peasi* | 5 | 1 | 1 | 2 | | 17 | 18 | 2 | 6 | | 9 | 43 | |
| *Synaptocochlea concinna* | 1 | 1 | 0 | 2 | | 6 | 32 | 0 | 12 | | 4 | 50 | |
| *Terebra guttata* | 3 | 1 | 4 | 1 | | 13 | 1 | 4 | 1 | | 9 | 19 | |
| *Tridentarius dentatus* | 4 | 7 | 6 | 0 | | 39 | 14 | 6 | 0 | | 17 | 59 | |
| *Turbonilla thaanumi* | 2 | 1 | 0 | 1 | | 6 | 4 | 0 | 3 | | 4 | 13 | |
| *Turbonilla varicosa* | 2 | 3 | 0 | 1 | | 2 | 4 | 0 | 4 | | 6 | 10 | |
| *Vexillum micra* | 3 | 2 | 1 | 1 | | 7 | 2 | 1 | 2 | | 7 | 12 | |
| *Zafra smithi* | 0 | 2 | 0 | 2 | | 0 | 20 | 0 | 9 | | 4 | 29 | |
| **TOTAL** | **72** | **65** | **34** | **58** | | **714** | **439** | **94** | **255** | | **229** | **1,502** | |
